# L-Arabinose Elicits Gut-Derived Hydrogen Production and Ameliorates Metabolic Syndrome in C57BL/6J Mice on High-Fat-Diet

**DOI:** 10.3390/nu11123054

**Published:** 2019-12-13

**Authors:** Lin Zhao, Yan Wang, Guanfei Zhang, Tiantian Zhang, Jing Lou, Jiankang Liu

**Affiliations:** Center for Mitochondrial Biology and Medicine, The Key Laboratory of Biomedical Information Engineering of Ministry of Education, School of Life Science and Technology, Xi’an Jiaotong University, Xi’an 710049, China; 13572504396@163.com (Y.W.); zgfei1021@126.com (G.Z.); sanjie1993@126.com (T.Z.); loujing16@foxmail.com (J.L.)

**Keywords:** L-arabinose, hydrogen, gut microbe, lipid metabolism, mitochondria, metabolic syndrome, high-fat-diet

## Abstract

Obesity and metabolic syndrome (MS) associated with excess calorie intake has become a great public health concern worldwide. L-arabinose, a naturally occurring plant pentose, has a promising future as a novel food ingredient with benefits in MS; yet the mechanisms remain to be further elucidated. Gut microbiota is recently recognized to play key roles in MS. Molecular hydrogen, an emerging medical gas with reported benefits in MS, can be produced and utilized by gut microbes. Here we show oral L-arabinose elicited immediate and robust release of hydrogen in mice in a dose-and-time-dependent manner while alleviating high-fat-diet (HFD) induced MS including increased body weight especially fat weight, impaired insulin sensitivity, liver steatosis, dyslipidemia and elevated inflammatory cytokines. Moreover, L-arabinose modulated gene-expressions involved in lipid metabolism and mitochondrial function in key metabolic tissues. Antibiotics treatment abolished L-arabinose-elicited hydrogen production independent of diet type, confirming gut microbes as the source of hydrogen. q-PCR of fecal 16S rDNA revealed modulation of relative abundances of hydrogen-producing and hydrogen-consuming gut microbes as well as probiotics by HFD and L-arabinose. Our data uncovered modulating gut microbiota and hydrogen yield, expression of genes governing lipid metabolism and mitochondrial function in metabolic tissues is underlying L-arabinose’s benefits in MS.

## 1. Introduction

The incidence of metabolic syndrome (MS) has been rising drastically all over the world in the past several decades along with the prevalence of modern lifestyle, especially a diet with excess calorie. MS encompasses a range of medical conditions like obesity, high blood sugar, dyslipidemia especially elevated serum triglycerides or cholesterol and reduced serum high-density lipoprotein (HDL), liver steatosis, high blood pressure, the list goes on. Insulin resistance or impaired insulin sensitivity sits at the pivot of those conditions and links to increased risk of developing type 2 diabetes, cardiovascular disease and some types of cancer, which makes it a severe concern of public health. Currently in China alone, it is estimated that about 440 million people live with prediabetes. Although changing lifestyle especially calorie restriction and increasing exercise is the recommended intervention, it is not effective for some due to physical incapability or the lack of will power. To prevent and alleviate MS without spoiling the pleasure of having meal with plenty of calories, functional food or nutritional ingredient from both traditional and novel resources are studied extensively in recent years. By far, non-caloric sugars have become one of the rising stars among nutraceuticals with great potential in anti-MS application with reported benefits in both animal experiments and human studies.

L-arabinose, a naturally occurring constituent of plant polysaccharides, usually extracted from vegetable gum, corn straw or beet, has gained considerable attention for its potential in anti-MS application recently. L-arabinose administration for 6 weeks reduces body weight, blood pressure, blood glucose, triglycerides, total cholesterol, serum insulin, serum TNF-α and serum leptin; and increases hepatic CPT1 and PDK4 mRNA level while decreases hepatic ACCα mRNA level in high-carbohydrate-high-fat-diet induced MS rats [1]. Polysaccharide from corn silk containing L-arabinose showed hypoglycemic and hypolipidemic effects on diabetic mice induced by high-fat-diet (HFD) and streptozotocin injection [2]. One mechanism underlying L-arabinose’s benefits in MS has been demonstrated as directly inhibiting intestinal sucrase activity both in vitro and in human [3], which explains why L-arabinose effectively lower blood glucose and insulin level with ingestion of high-sucrose food or beverages but is unable to account for L-arabinose’s benefits in MS in general. Therefore, more studies are urgently called to illuminate the mechanisms underlying L-arabinose’s effects in HFD models, considering fat being a major source of energy intake in reality.

Gut microbiota has been recognized to play a pathogenic role in the development of MS in both humans and animal models in the last few decades, as it affect host glucose and lipid metabolism, hepatic fatty storage, insulin sensitivity and systemic inflammation [4,5,6,7]; diet exerts important influence on gut microbial profile and HFD-induced alterations in gut microbial composition is thought to be a driving force for obesity and chronic disease risk [8,9,10]; manipulating gut microbial ecology, such as fecal microbiota transplant, has therapeutic potential in treating obesity and metabolic syndrome [11]. Gut microbes generate a diverse metabolite repertoire that is proposed to mediate the effects of gut microbiome on host health and disease, some metabolites can promote metabolic benefits on body weight and glucose control, such as short-chain fatty acids like acetate, propionate and butyrate [12,13,14]. Importantly, molecular hydrogen(H_2_), with a recent recognition of being a novel biologically-active gas exhibiting antioxidant, antiapoptotic, anti-inflammatory, cytoprotective and signaling properties and having great potential as a preventive and therapeutic medical gas[15,16,17], can be generated by fermentative metabolism in anaerobic ecosystems [18,19]. Molecular hydrogen has long been identified as a main component of intestinal gas in humans and respiratory hydrogen gas excretion can indicate intestinal hydrogen gas production [20]. Some saccharides have been reported to increase hydrogen gas production through gut fermentation, such as acarbose [21], fructo-oligosaccharide and galactosyl-sucrose [22]. Now it is well accepted that microbial molecular hydrogen-cycling—composed of hydrogen production from fermentation of non-digested carbohydrate and hydrogen reoxidation primarily by sulfate reduction, acetogenesis and methanogenesis—is central to gut microbial composition and metabolic homeostasis and to host health outcomes [23]. Inadequate production of H_2_ by gut microbiota has been linked to diseases [24]. Drinking H_2_-water improves obesity, diabetes and MS by stimulating energy metabolism in db/db mice and also significantly alleviates HFD-induced fatty liver in wild-type mice [25]. We have also shown that hydrogen supplementation by coral calcium hydride administration, a solid molecular hydrogen carrier, prevents obesity and hepatic steatosis in rats on HFD while improving mitochondrial function impaired by HFD and inducing phase enzymes in liver [26]. Mitochondrial dysfunction is closely related to insulin resistance and metabolic syndrome [27,28]; molecular hydrogen has been proposed to be a novel antioxidant that can reduce oxidative stress and have the potential to improve mitochondrial diseases [29]; transport of molecular hydrogen to mitochondria has been discussed[30]. Besides, hydrogen was recently reported to regulates gene expression by modifying the free radical chain reaction-dependent generation of oxidized phospholipid mediators [31]. However, whether and how L-arabinose affect gut microbes, intestinal hydrogen gas production, mitochondrial function, gene expression profile in metabolic tissues while alleviating MS remain largely unknown. Here, we aimed to address these points in HFD-induced MS model in mice.

We report for the first time that L-arabinose gavage elicited immediate and robust release of hydrogen in mice in a dose-and-time-dependent manner, which can be abolished by antibiotics treatment regardless of diet type; long-term L-arabinose administration effectively reduced body weight gain especially fat weight, improved insulin sensitivity, alleviated liver steatosis, systemic dyslipidemia and inflammation in mice on HFD. Further analysis revealed that L-arabinose altered gene expression governing lipid metabolism and mitochondrial function in key metabolic tissues. Moreover, q-PCR of fecal 16S rDNA indicated both HFD and L-arabinose modulated relative abundances of hydrogen-producing and hydrogen-consuming gut microbes as well as probiotics. Collectively, our data provide a plausible explanation linking gut microbes, intestinal hydrogen yield, lipid metabolism gene expression and mitochondrial function in metabolic tissues together to lay ground for thoroughly describing and deciphering L-arabinose’s working mechanism underlying its efficacy in MS associated with HFD. This novel perspective shall pave the way for a better application of L-arabinose in preventing or treating MS.

## 2. Materials and Methods

### 2.1. Reagents

Antibodies list: CPT1A (A5307, ABclonal, Wuhan, China), UCP1 (A5857, ABclonal), UCP3(A16996, ABclonal), PPARα (sc-9000, Santa Cruz, Dallas, TX, USA), PPARγ (2435S, CST, Danvers, MA, USA), FAS (3189S, CST), SREBP1 (Sc13551, Santa Cruz), PGC-1α (TA319007, Origene, Rockville, MD, USA), NDUFS3 (459130, Invitrogen, Waltham, MA, USA), SDHB (459230, Invitrogen), UQCRC1 (459140, Invitrogen), COX4 (459600, Invitrogen), ATP Synthase Subunit Alpha (459240, Invitrogen), SOD2 (sc-137254, Santa Cruz), β-Actin (3700S, CST), α-Tubulin (3873S, CST).

Mice diet was provided by SLAC Laboratory Animal Co. Ltd. (Shanghai, China). Insulin was purchased from Nove Nordisk A/S. The blood glucose meter and blood glucose test strip were both purchased from ROCHE (ACCU-CHEK Active).

The Reverse Transcription System kit was purchased from Promega; SYBR green was purchased from Takara; polymerase chain reaction (PCR) primers were synthesized by Beijing Qingke biotechnology Co. Ltd. Nitrocellulose membranes used in W.B. were purchased from PerkinElmer Life Sciences. Other reagents used in this study were purchased from Sigma (St. Louis, MO, USA).

### 2.2. Animals and Procedures

C57BL/6 male mice were purchased from the SLAC Laboratory Animal Co. Ltd. (Shanghai, China) and were housed in a temperature- (22–28 °C) and humidity- (60%) controlled animal room and maintained under a 12-h light/dark cycle with free access to food and water throughout the experiments. After 1 week of acclimatization, mice were first measured for background hydrogen production rate and then were randomly assigned into different groups. CD, mice fed a chow diet (CD, 10% fat content) with vehicle gavage; HFD, mice fed a high-fat diet (HFD, 60% fat content) with vehicle gavage; HFD+A-L, mice fed a HFD with low-dose of L-arabinose gavage (500 mg/kg/day); HFD+A-H, mice fed a HFD with high dose of L-arabinose gavage (5 g/kg/day). The compositions and formulas of mice diets are presented in detail in Table 1. During the experiment, hydrogen production capacity was assessed at indicated time; mice were fasted overnight before OGTT (oral glucose tolerance test) or ITT (insulin tolerance test) at indicated time; mice feces were freshly collected at indicated time. Body weight and food intake were monitored and recorded once every week. At the end of the experiment, mice were fasted overnight and euthanized before dissection.

For the second batch of mice, a combination of antibiotics water solution was administered via oral gavage for the first 3 days at high concentration to effectively suppress gut microbes and then via supplementation into drinking water at low concentration to avoid reduce in water intake, because L-arabinose need to be administered via oral gavage to guarantee the precision of dose.

The animal experiments protocol and procedure were approved by School of Life Science and Technology, Xi’an Jiaotong University (ethical approval code: Xi’an Jiaotong University Life Science (2016) No. 27). All procedures and performances involving experimental animals are in accordance with the Guide for the Care and Use of Laboratory Animals (8th edition) and other approved guidelines.

### 2.3. Fecal DNA Extraction

Mice fecal samples were freshly collected at 1-week, 1-month and 3-month time-points, notably mice from the same cage were kept together in a sterilized empty box for 1–2 h to collect one sample. Three samples were collected for each experimental group at each time-point. Total gDNA was extracted with a commercial gDNA extraction kit (QIAamp Fast DNA Stool Mini Kit, QIAGEN, #51604) according to the manufacture’s instruction.

### 2.4. Histopathologic Analysis

For histological analysis, freshly collected liver tissue were fixed in 10% formalin, sectioned and stained with H & E method.

### 2.5. Measurements of Serum Parameters

After the mice were sacrificed, orbital blood sampling was performed and the serum was separated by centrifugation (1200× *g*, 10 min). All serum parameters were measured by using a commercially available ELISA (enzyme-linked immunosorbent assay) kit or biochemical assay kit (Jiancheng, Nanjing, China) according to the manufacturer’s instructions.

### 2.6. Liver Lipid Profile Measurement

A small portion of liver tissue were collected and homogenized in ice-cold phosphate buffered saline (PBS). After centrifugation (1000× *g*, 10 min), the supernatant was collected for analysis. Triglyceride (TG) and total cholesterol (TC) levels were analyzed using commercial clinical diagnosis kits according to the manufacturer’s instructions (Jiancheng, Nanjing, China).

### 2.7. Quantitative Real-Time PCR

To determine the relative abundance of certain groups of bacteria in mice feces, gDNA was used as a template for quantitative real-time PCR reaction with SYBR Green Master Mix (TaKaRa) and genus-/species-/groups-specific primer-sets targeting 16S Ribosomal RNA gene; amplification of universal bacterial 16S rRNA gene was used as internal reference. The primer-sets for quantification of specific bacterial groups are listed in Table 2.

To relatively measure tissue mRNA transcripts level, total RNA was extracted from 30 mg of tissue using TRIzol reagent (Invitrogen) according to the manufacturer’s protocol and RNA quality was monitored by reading OD260/OD280 ratio. For each sample, 2 μg of RNA was reverse transcribed into cDNA. Quantitative PCR was performed using SYBR Green Master Mix (TaKaRa) with gene-specific primers (listed in Table 3).

### 2.8. SDS-PAGE and Western-Blot

Indicated tissue samples were homogenized and lysed with Western and IP (immunoprecipitation) lysis buffer (Beyotime) and were centrifuged at 13,000× *g* for 20 min at 4 °C. The supernatants were collected and measured for protein concentrations with a BCA (bicinchoninic acid assay) protein assay kit before electrophoresis using 10% SDS-PAGE gels to separate proteins in samples. After western-blot, chemiluminescent detection was performed to obtain signals for individual proteins.

### 2.9. Isolation of Mitochondria and Determination of Mitochondrial Electron-Transport-Chain Complexes Activities

The procedures used for mitochondria isolation from tissues by differential centrifugation method, along with the assay methods for determination of mitochondrial electron-transport-chain complexes activities (reduced nicotinamide adenine dinucleotide (NADH)-ubiquinone reductase (Complex I), succinate-CoQ oxidoreductase (complex II), ubiquinol cytochrome c reductase (complex III) and Mg2+-ATPase (Complex V)) were described previously [41].

### 2.10. Statistical Analysis

GraphPad Prism software (GraphPad Software, Version 8.0.2, San Diego, CA, USA) was used to perform statistical analysis. The data are presented as the mean ± SEM. *P* values were calculated by one-way ANOVA with multiple comparisons or the unpaired Student’s *t*-test, as appropriate and indicated in the figure legends.

## 3. Results

### 3.1. Oral L-Arabinose Elicits Hydrogen Production in Mice in a Time-and-Dose-Dependent Manner

**Figure 1:**
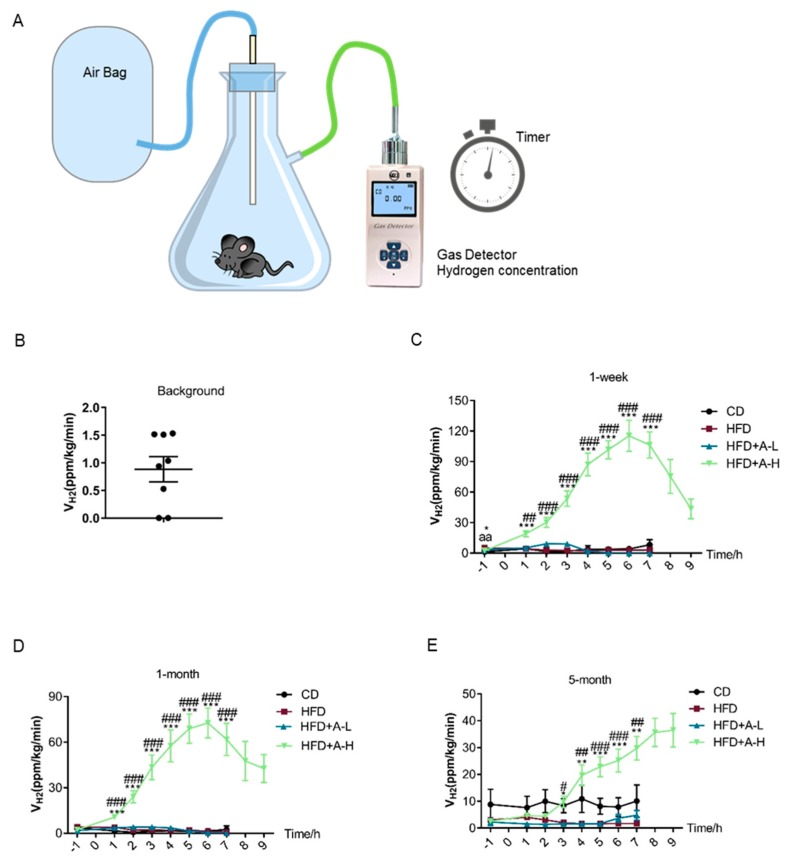
Oral L-arabinose elicits hydrogen production in mice in a time-and-dose-dependent manner. (**A**) Schematic illustration of the self-built device for detection of hydrogen production in mice. (**B**) Background hydrogen production rate of experimental mice (*n* = 32). (**C**–**E**) Hydrogen production rate of mice were measured 1 h before and every hour from 2 to 9 h after L-arabinose gavage at 1-week, 1-month and 5-month time-points, respectively; L-arabinose was administered via oral gavage at 0-hour time-point; CD (chow diet), HFD (high fat diet), HFD+A-L (HFD + 500 mg/kg ARA (arabinose), low-dose), HFD-A-H (HFD + 5 g/kg ARA, high dose), *n* = 8. Values are mean ± SEM. Statistical analyses were conducted using One-way ANOVA followed by Tukey’s Multiple Comparison Test. ^a^ *p* < 0.05, ^aa^ *p* < 0.01, ^aaa^ *p* < 0.001, HFD vs. control; * *p* < 0.05, ** *p* < 0.01, *** *p* < 0.001, HFD+A-L/HFD+A-H vs. HFD; ^#^ *p* < 0.05, ^##^ *p* < 0.01, ^###^ *p* < 0.001, HFD+A-H vs. HFD+A-L.

To determine the hydrogen production capacity of mice, we first built a self-designed simple device mainly composed of a suction flask, an air bag, a timer and a gas detector that reads hydrogen concentration in the air, as illustrated in Figure 1A. Notably, two or four mice were put together into the flask at the same time to avoid any irritation to experimental animals caused by isolation in relatively narrow space. All possible leaking connection sites were sealed with Parafilm to guarantee the airtightness of the system before starting the measurement. After the mice were in place and the system were sealed, we detected the air hydrogen concentration in the system every 10 min within a 30-minute duration and then calculated the hydrogen production velocity as hydrogen concentration accumulation rate divided by body weight of mice accordingly. Before the random assignment of experimental groups for mice, we checked the background hydrogen production capacity of those mice at random time of day and found extremely low level of hydrogen release which can be neglected as shown in Figure 1B.

To investigate whether L-arabinose can induce the production of hydrogen gas in mice and exerting beneficial effects on MS, we fed 8-week old male C57BL/6J mice either a chow diet (CD) (10% calories from fat) or a high-fat-diet (HFD) (60% calories from fat) to establish diet-induced MS model; the mice on HFD were randomly assigned into three groups and given water, low-dose of L-arabinose (500 mg/kg body weight) or high dose of L-arabinose (5 g/kg body weight) by oral gavage once daily. During the animal experiment, we determined hydrogen production capacity of the mice at different time, 1-week, 1-month and 5-month, respectively. Briefly, the gavage administration time was set as time-point “0”, the determination of hydrogen production capacity was conducted once one hour before gavage at time-point “−1” and then once every hour after gavage until the decline of the curve denoting hydrogen production capacity occurred. In all three measurements, at 1-week, 1-month and 5-month time-point, respectively, we observed that high dose of L-arabinose gavage could immediately elicit a dramatic increase of hydrogen production velocity in mice which lasted up to 9 h (Figure 1C–E). Interestingly, the peak value of the hydrogen production velocity curve at 1-week time was the highest among the three times of measurement and gradually dropped along with time; besides, the peak time of the hydrogen production velocity curve was around 6 h after gavage both in 1-week and in 1-month time-point but seemed to get postponed in 5-month time-point (Figure 1C–E). In addition, only high dose of L-arabinose exhibited the effects while low-dose of L-arabinose failed to elicit any release of hydrogen production in mice (Figure 1C–E). Together, these results clearly show that oral L-arabinose elicits hydrogen production in mice in a time-and-dose-dependent manner.

### 3.2. L-arabinose Reduces Body Weight, Liver Weight to Body Weight Ratio, Fat Weight to Body Weight Ratio and Serum Leptin Level in Mice on HFD Without Affecting Calorie Intake

**Figure 2:**
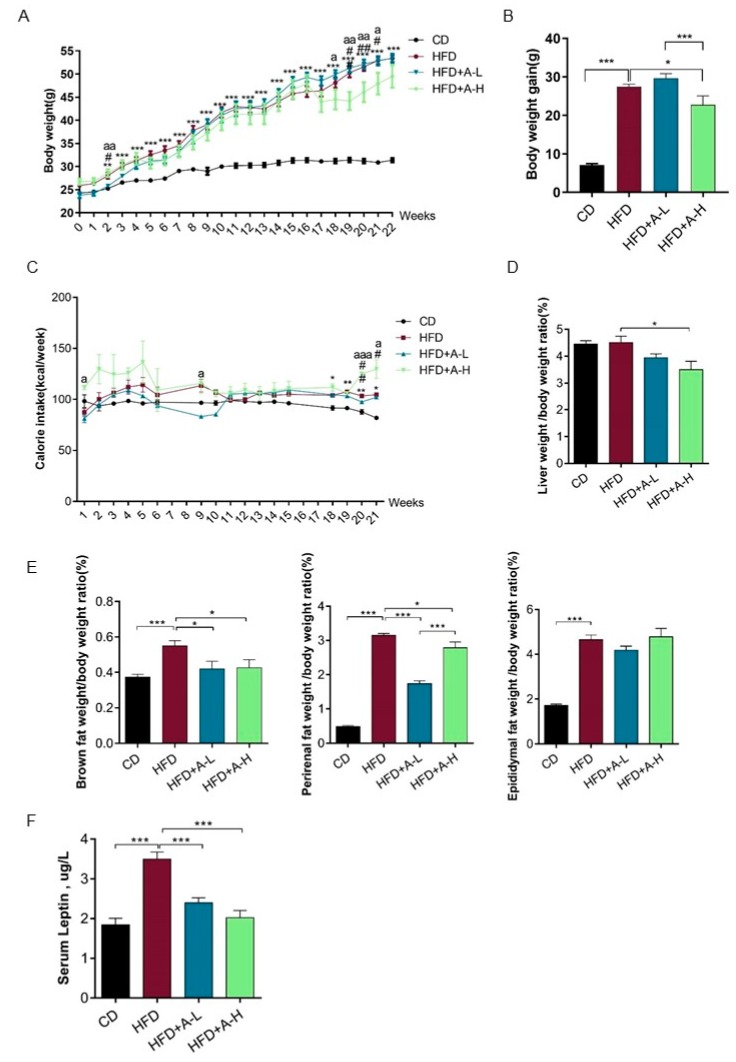
L-arabinose reduces body weight, liver weight, fat weight and serum leptin level in mice on HFD without affecting food intake. (**A**) Body weight of mice throughout the experimental course. (**B**) Body weight gain of mice throughout the experimental course. (**C**) Calorie intake of mice throughout the experimental course. (**D**) Liver weight of mice at the endpoint of the experiment. (**E**) Brown fat weight, perirenal fat weight and epididymal fat weight of mice at the endpoint of the experiment. (**F**) Serum leptin level at the endpoint of the experiment. (**A**–**C**), CD *n* = 15; HFD, *n* = 12; HFD+A-L, *n* = 7; HFD-A-H, *n* = 6; (**D**,**E**), CD, *n* = 15; HFD, *n* = 12; HFD+A-L, *n* = 6; HFD-A-H, *n* = 6 ; (**F**), CD, *n* = 9; HFD, *n* = 9; HFD+A-L, *n* = 6; HFD-A-H, *n* = 6. Values are the mean ± SEM. Statistical analyses were conducted using One-way ANOVA followed by Tukey’s Multiple Comparison Test. (**A**,**C**) * *p* < 0.05, ** *p* < 0.01, *** *p* < 0.001, HFD vs. CD; ^#^ *p* < 0.05, ^##^ *p* < 0.01, ^###^ *p* < 0.001, HFD+ARA-L/HFD+ARA-H vs. HFD; ^a^ *p* < 0.05, ^aa^ *p* < 0.01, ^aaa^ *p* < 0.001, HFD+ARA-L vs. HFD+ARA-H. B, D, E &F: * *p* < 0.05, ** *p* < 0.01, *** *p* < 0.001.

Besides the observation of hydrogen production in mice, we also found that high dose of L-arabinose significantly reduced the body weight gain drastically elevated by HFD without affecting calorie intake in a 22-week time course (Figure 2A–C). Further, when the mice were sacrificed at the endpoint of the experiment, we found that HFD-induced a substantial increase in the weight of the major tissues involved in lipid anabolism, including liver, brown fat, perirenal fat and epididymal fat; when calculating the ratio of tissue weight to body weight, we found that a high dose of L-arabinose significantly reduced the ratio of liver weight to body weight in mice on HFD, both a high dose and low-dose of L-arabinose significantly reduced brown fat weight to body weight ratio and perirenal fat weight to body weight ratio that were increased by HFD, while both high dose and low-dose of L-arabinose failed to affect epididymal fat weight to body weight ratio that was increased by HFD (Figure 2D,E). These observations indicate that L-arabinose have a major impact on fat storage in HFD models. Leptin is secreted by adipose tissue and acts to regulate energy expenditure and appetite as body fat stores increase. We looked at serum leptin level and found that serum leptin level was indeed raised by HFD but was effectively restored to a similar level as in chow diet group by high dose or low-dose of L-arabinose administration (Figure 2F). The changes of serum leptin level exhibit a similar pattern as that of brown fat weight to body weight ratio among experimental groups, suggesting a potential key role of brown adipose tissue in leptin secretion or response. These findings indicate that L-arabinose effectively reduces body weight gain, especially the weight of tissues intimately involved in lipid anabolism and serum leptin level, which were all increased robustly by HFD.

### 3.3. L-Arabinose Improves Glucose Homeostasis in Mice on HFD

**Figure 3:**
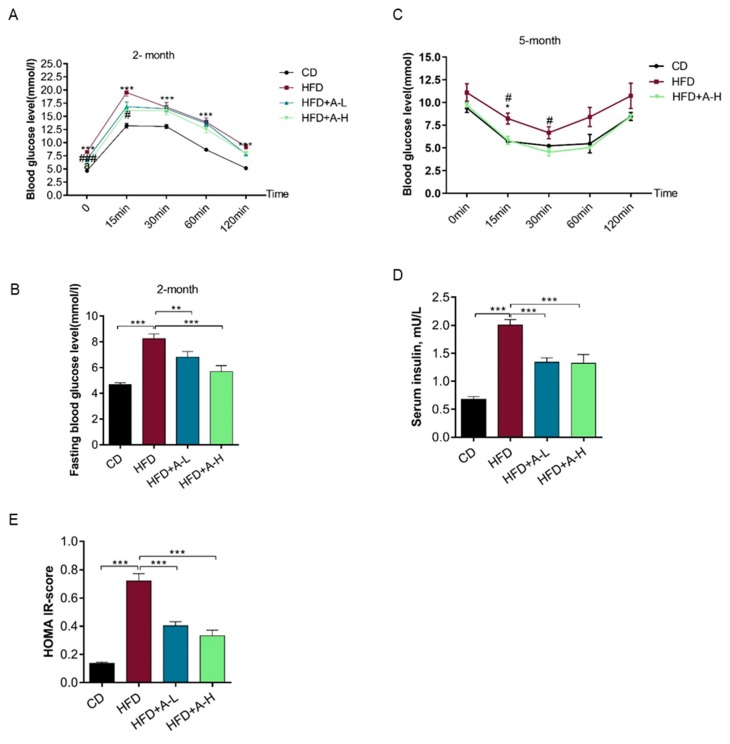
L-arabinose improves glucose homeostasis in mice on HFD. (**A**) OGTT of mice at 2-month time-point. (**B**) fasting blood glucose level at 2-month time-point. (**C**) ITT of mice at 5-month time-point. (**D**) fasting serum insulin at endpoint of the experiment. (**E**) Calculated HOMA IR-score. (**A**,**B**), CD, *n* = 15; HFD, *n* = 12; HFD+A-L, *n* = 6; HFD-A-H, *n* = 6; (**C**), CD, *n* = 3; HFD, *n* = 3; HFD-A-H, *n* = 3; (**D**,**E**), CD , *n* = 9; HFD, *n* = 9; HFD+A-L, *n* = 6; HFD-A-H, *n* = 6. Values are the mean ± SEM. Statistical analyses were conducted using One-way ANOVA followed by Tukey’s Multiple Comparison Test. (**A**,**D**) * *p* < 0.05, ** *p* < 0.01, *** *p* < 0.001, HFD vs. CD; ^#^ *p* < 0.05, ^##^ *p* < 0.01, ^###^ *p* < 0.001, HFD vs. HFD+ARA-L/HFD+ARA-H. (**B,C,E–G**) * *p* < 0.05, ** *p* < 0.01, *** *p* < 0.001.

At two-month time-point of the experiment, we checked glucose homeostasis of the mice by OGTT. Compared to mice on CD, the glucose tolerance of mice on HFD was substantially impaired and was significantly improved by high dose of L-arabinose (Figure 3A). Importantly, fasting blood glucose level was lifted by HFD and was lowered by both low-dose and high dose of L-arabinose treatment at two-month time (Figure 3B). Before sacrificing the mice at 5-month time, we did ITT and found that insulin sensitivity of mice on HFD was compromised compared to mice on CD and high dose of L-arabinose treatment successfully recovered insulin sensitivity of mice on HFD (Figure 3C); meanwhile, fasting serum insulin was elevated by several folds in mice on HFD compared with mice on CD and was effectively reduced by either high dose or low-dose of L-arabinose treatment (Figure 3D). Accordingly, we calculated HOMA IR (Homeostatic Model Assessment for Insulin Resistance) score and it clearly shows that HFD could lead to insulin resistance that is corrected by either high dose or low-dose of L-arabinose treatment (Figure 3E). These results indicate that HFD results in impaired glucose homeostasis and insulin resistance which can be effectively relieved by L-arabinose.

### 3.4. L-arabinose Effectively Alleviates Liver Steatosis As Well As Restores Serum Lipid Profile and Inflammatory Parameters in Mice on HFD

**Figure 4:**
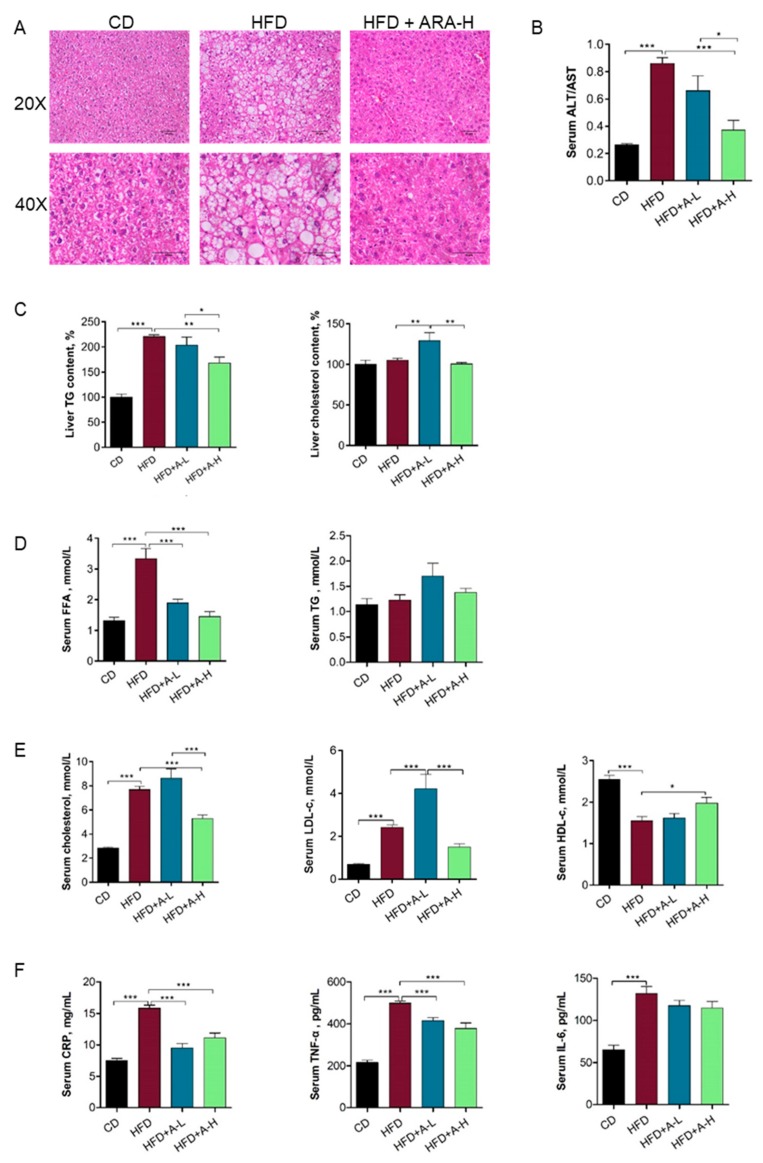
L-arabinose effectively alleviates liver steatosis as well as restores serum lipid profile and inflammatory parameters in mice on HFD. (**A**) Representative image of H & E (Hematoxylin and eosin) staining of liver section (*n* = 3). (**B**) Serum ALT/AST (alanine transaminase/aspartate transaminase) ratio. (**C**) Relative liver TG (triglycerides) content and liver cholesterol content. (**D**) Serum FFA (free fatty acid) and TG level. (**E**) Serum cholesterol level, LDL-c (low-density lipoprotein cholesterol) level and HDL-c (high-density lipoprotein cholesterol) level. (**F**) Serum CRP (C-reactive protein) level, TNF-α (Tumor Necrosis Factor Alpha) level and IL-6 (Interleukin 6) level. (**B,D**–**F**), CD, *n* = 9; HFD, *n* = 9; HFD+A-L, *n* = 6; HFD-A-H, *n* = 6. C, for cholesterol measurement, CD, *n* = 9; HFD, *n* = 9; HFD+A-L, *n* = 6; HFD-A-H, *n* = 6; for TG measurement, CD, *n* = 9; HFD, *n* = 9; HFD+A-L, *n* = 6; HFD-A-H, *n* = 5. Values are the mean ± SEM. Statistical analyses were conducted using One-way ANOVA followed by Tukey’s Multiple Comparison Test. * *p* < 0.05, ** *p* < 0.01, *** *p* < 0.001.

At five-month time-point, mice on HFD developed evident liver steatosis but high dose of L-arabinose completely abolished the accumulation of lipid droplet in the liver induced by HFD as shown by H & E staining of liver section (Figure 4A). Further biochemical measurements of liver homogenates revealed elevated liver TG (triglyceride) content in mice on HFD and significant decrease of liver TG by high dose of L-arabinose (Figure 4C), corroborating the effect of L-arabinose on alleviating liver steatosis. Liver cholesterol content was similar among CD group, HFD group and high dose of L-arabinose treated HFD group but was higher in low-dose of L-arabinose treated HFD group (Figure 4C), suggesting that L-arabinose might affect cholesterol metabolism. Biochemical analysis of serum showed that fasting ALT (alanine aminotransferase) to AST (aspartate aminotransferase) ratio was boosted by several folds in mice on HFD compared with mice on CD and both low-dose and high dose of L-arabinose significantly decreased ALT/AST ratio in mice on HFD, with high dose of L-arabinose reducing ALT/AST ratio in mice on HFD to a comparable level as that in mice on CD (Figure 4B). These data together indicate that L-arabinose protects from fatty liver associated hepatocellular damage or toxicity induced by HFD.

We then checked fasting serum lipid profile in these mice at five-month time-point. HFD resulted in elevated serum level of FFA (free fatty acid) and both low-dose and high dose of L-arabinose significantly lowered serum FFA in mice on HFD to a similar level as in mice on CD; in contrast, serum TG level was not changed among all experimental groups (Figure 4D). Serum total cholesterol level in mice on HFD was higher than in mice on CD but was significantly reduced by high dose of L-arabinose; serum LDL-c (Low-density lipoprotein cholesterol) was increased in mice on HFD compared with mice on CD and was further increased by low-dose of L-arabinose treatment but was substantially reduced by high dose of L-arabinose treatment in mice on HFD; serum HDL-c (High-density lipoprotein cholesterol) was decreased in mice on HFD compared with mice on CD but was effectively restored by high dose of L-arabinose treatment (Figure 4E). These observations suggest that L-arabinose have a role in modulating systemic cholesterol homeostasis. Overall, these data indicate that L-arabinose is efficacious in restoring altered lipid profile induced by HFD and is potentially beneficial in lowering cardiovascular risk associated with MS.

Since MS is also recognized as an inflammatory disorder [42], we checked several systemic inflammation markers in the serum. CRP (C-reactive protein), produced by the liver in response to inflammation, was found elevated in mice on HFD and was effectively reduced to a similar level as mice on CD by both low-dose and high dose of L-arabinose treatment; serum TNF-α (tumor necrosis factor alpha) level alterations exhibited similar pattern to serum CRP; serum IL-6 (interleukin 6) was also elevated in mice on HFD compared to mice on CD but was not altered by either low-dose or high dose of L-arabinose treatment (Figure 4F). The changes of serum CRP and TNF-α reflect heightened level of systemic inflammation in mice on HFD that was successfully corrected by both low-dose and high dose of L-arabinose treatment, which was not contradicted by the observed alterations of IL-6 as it can act as both a pro-inflammatory cytokine and an anti-inflammatory myokine.

### 3.5. L-arabinose Modulates mRNA and Protein Expression Pattern of Selected Genes Involved in Lipid Metabolism in Key Lipid-Metabolizing Tissues in Mice on HFD

The above results that L-arabinose effectively reduced body weight especially fat weight, alleviated liver steatosis and improved serum lipid profile in mice on HFD have clearly shown that L-arabinose can profoundly modulate systemic lipid metabolism altered by HFD. To investigate the mechanisms underlying L-arabinose’s beneficial effects on MS induced by HFD, we checked both mRNA and protein expression levels of some pivotal genes participating in lipid anabolism or catabolism in several tissues that are metabolically active or playing key roles in systemic lipid metabolism.

**Figure 5:**
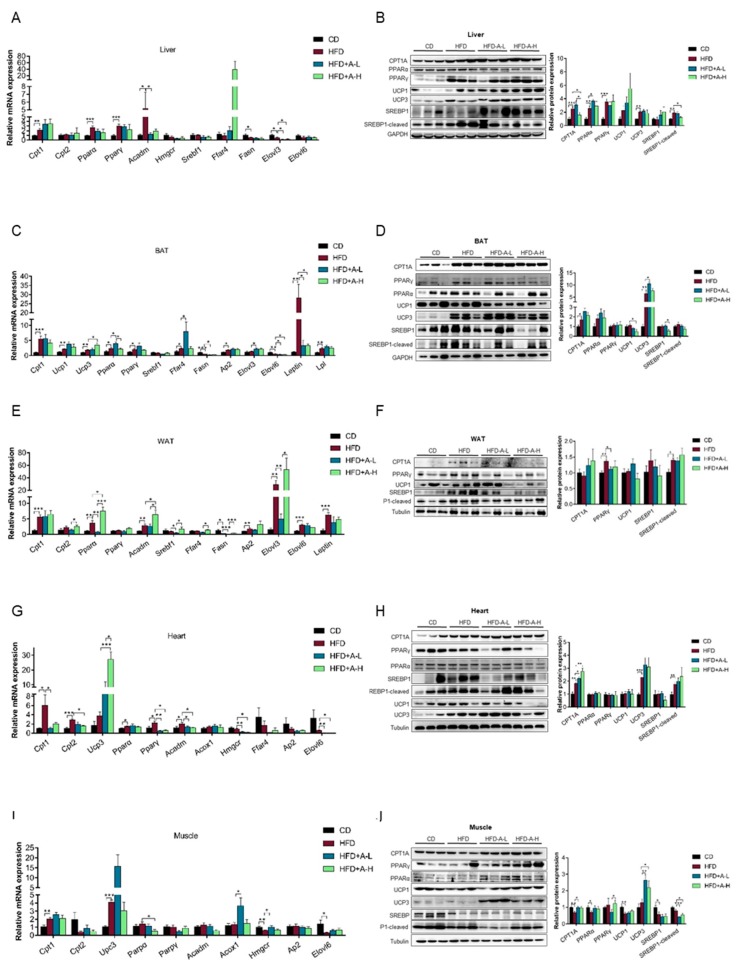
L-arabinose modulates mRNA and protein expression pattern of selected genes involved in lipid metabolism in key lipid-metabolizing tissues in mice on HFD. Relative mRNA expression of genes involved in lipid metabolism in liver tissue (**A**), brown adipose tissue (BAT) tissue (**C**), WAT (white adipose tissue) tissue (**E**), heart tissue (**G**) and muscle tissue (**I**) were detected by real-time q-PCR. Protein expression of key molecules involved in lipid metabolism in liver tissue (**B**), BAT tissue (**D**), WAT tissue (**F**), heart tissue (**H**) and muscle tissue (**J**) were detected by SDS-PAGE and W.B. and then quantified by densitometry analysis. (**A**), CD, *n* = 6; HFD, *n* = 6; HFD+A-L, *n* = 6; HFD+A-H, *n* ≥ 3; (**B**), CD, *n*=6; HFD, *n* = 6; HFD+A-L, *n* = 6; HFD-A-H, *n* = 6; (**C**), CD, *n* = 6; HFD, *n* = 6; HFD+A-L, *n* = 5; HFD-A-H, *n* = 4; (**D**), CD, *n* = 5; HFD, *n* = 6; HFD-A-H/HFD+A-L, *n* = 4; (**E**), CD, *n* = 6; HFD, *n* = 6; HFD+A-L, *n* ≥ 5; HFD-A-H, *n* = 4; (**F**), CD, *n* = 6; HFD, n = 6; HFD+A-L, *n* = 6; HFD-A-H, *n* = 4; (**G**), CD, *n* ≥ 5; HFD, *n* ≥ 5; HFD+A-L, *n* = 6; HFD-A-H, *n* = 3; (**H**), CD, *n* = 6; HFD, *n* = 6; HFD+A-L, *n* = 6; HFD-A-H, *n* = 3; (**I**), CD, *n* = 6; HFD, *n* = 6; HFD+A-L, n = 5; HFD-A-H, *n* = 3; (**J**), CD, *n* = 6; HFD, *n* = 6; HFD+A-L, *n* = 6; HFD-A-H, *n* = 6. Values are the mean ± SEM. Statistical analyses were conducted using two-tailed unpaired *t*-test analysis. * *p* < 0.05, ** *p* < 0.01, *** *p* < 0.001.

In the liver, HFD significantly increased the levels of mRNA transcripts of genes involved in lipid catabolism, including *Cpt1* (carnitine palmitoyl transferase 1) which is crucial to fatty acid transportation into the mitochondria, *Pparα* (peroxisome proliferator-activated receptor alpha) and *Pparγ* (peroxisome proliferator-activated receptor gamma) which play vital role in regulating expression of fatty acid utilization genes and *Acadm* (acyl-Coenzyme A dehydrogenase, medium chain) which catalyzes the initial step of fatty acid beta-oxidation(Figure 5A). HFD also significantly increased protein levels of CPT1A, PPARα and PPARγ, as well as UCP3 (uncoupling protein 3) which creates proton leaks across the inner mitochondrial membrane thereby uncouples oxidative phosphorylation and dissipate energy as heat (Figure 5B), suggesting that HFD increases fatty acid utilization and thermogenic respiration. The stimulation of lipid catabolism by HFD is expected due to sufficient supply of fat. Notably, L-arabinose treatment, low-dose, effectively restored mRNA level of *Acadm* but did not affect mRNA expression of the rest of tested genes in lipid catabolism (Figure 5A), high dose of L-arabinose reduced HFD-induced increase of CPT1A protein level and low-dose of L-arabinose further increased PPARα protein level (Figure 5B), suggesting that the alleviation of HFD-induced fatty liver, hyperlipidemia and obesity by L-arabinose might not be mediated by enhancing fatty acid utilization or thermogenesis in the liver. On the other hand, HFD significantly decreased the levels of mRNA transcripts of genes involved in lipid anabolism, including *Fasn* (fatty acid synthase), which catalyzes the formation of long-chain fatty acids and *Elovl3* (ELOVL fatty acid elongase 3), which catalyzes the first and rate-limiting reaction of the long-chain fatty acids elongation cycle (Figure 5A). The inhibition of long-chain fatty acid synthesis by HFD is also expected due to sufficient supply of long-chain fatty acids. Importantly, both low-dose and high dose of L-arabinose further decreased mRNA level of *Elovl3* (Figure 5A), suggesting that the alleviation of HFD-induced metabolic syndrome by L-arabinose might be mediated by suppressing long-chain fatty acid synthesis in liver. We also found that HFD increased protein level of cleaved SREBP1(sterol regulatory element binding protein/transcription factor 1) (Figure 5B), the activated form of transcriptional activator SREBP1 regulating lipid homeostasis as well as cholesterol synthesis pathway; and L-arabinose treatment restored it (Figure 5B); whereas the mRNA level of *Srebf1* remained similar among four groups (Figure 5A). We speculate that increasing and recovering the cleavage of SREBP1 might be one of the mechanisms underlying the disruption and the restoration serum cholesterol level in mice by HFD and L-arabinose treatment, respectively (Figure 4E).

In BAT (brown adipose tissue), HFD significantly increased the levels of mRNA transcripts of genes involved in lipid catabolism, too, including *Cpt1*, *Ucp1, Ucp3, Pparα, Pparγ* and *Lpl* (lipoprotein lipase) which catalyzes the hydrolysis of triglycerides from circulating chylomicrons and very low-density lipoproteins, while L-arabinose treatment further increased mRNA levels of *Ucp3* and *Pparα* (Figure 5C); protein levels of CPT1A and UCP3 exhibited similar pattern (Figure 5D). These data suggest that HFD-induced increased lipid utilization and energy dissipation as heat in BAT, probably due to overload of fat supply and L-arabinose might further enhance it by upregulating UCP3. In addition, HFD significantly decreased the levels of mRNA transcripts of genes involved in lipid anabolism, including *Fasn* and *Elovl6 and* L-arabinose significantly further decreased them (Figure 5C), suggesting that HFD-induced inhibition of fatty acid synthesis and the alleviation of HFD-induced metabolic syndrome by L-arabinose might be mediated by suppressing long-chain fatty acid synthesis in BAT too. Notably, although both mRNA level of *Srebf1* and protein level of cleaved SREBP1 were not altered among four groups (Figure 5C,D), high dose of L-arabinose significantly decreased protein level of SREBP1 (Figure 5D), suggesting L-arabinose could possibly modulate protein stability of SREBP1 maybe mediated by post-translational modification or protein-protein interaction, which might also contribute to L-arabinose’s alleviation of HFD-induced MS. Besides, HFD increased and low-dose of L-arabinose treatment further increased mRNA levels of *Ffar4* (free fatty acid receptor 4 or GPR120, G-protein coupled receptor 120) (Figure 5C), which encodes a G-protein coupled receptor for medium and long-chain free fatty acids that is reported to mediate potent insulin sensitizing and anti-diabetic effects by repressing macrophage-induced tissue inflammation[43], indicating that transcription of *Ffar4* is adaptively upregulated in BAT of mice on HFD and suggesting that L-arabinose’s beneficial effects on MS could be mediated via activating expression of *Ffar4*. HFD also upregulated mRNA level of *Ap2* (adipocyte protein 2 or Fabp4, Fatty acid binding protein) (Figure 5C), which is a lipid transport protein in adipocytes and is involved in brown and white fat cell differentiation, cholesterol homeostasis and positive regulation of inflammatory response, implying a possible role of increased expression of *Ap2* underlying the increase of WAT weight and the elevation of serum pro-inflammatory cytokines in mice on HFD. Finally, mRNA level of leptin was dramatically boosted by HFD and was effectively restored by both low-dose and high dose of L-arabinose treatment in BAT (Figure 5C), which corresponds well to the changing pattern of serum leptin level (Figure 2F), suggesting that BAT-derived leptin contributes a major part to systemically circulating leptin.

In WAT (white adipose tissue), in regard to lipid catabolism, HFD significantly increased the levels of mRNA transcripts of *Cpt1, Pparα* and *Acadm;* while low-dose of L-arabinose recovered mRNA level of *Pparα* and high dose of L-arabinose further increased mRNA levels of *Pparα* and *Acadm* (Figure 5E); protein level of PPARγ was increased by HFD and was recovered by L-arabinose treatment (Figure 5F). These data suggest that HFD might increase fatty acid utilization by upregulating key molecules in fatty acid beta-oxidation and thermogenesis in mitochondria of WAT and L-arabinose could further boost it which may contribute to its alleviation of HFD-induced MS. Simultaneously, in regard to lipid anabolism, HFD significantly decreased the levels of mRNA transcripts of *Fasn* but substantially increased the levels of mRNA transcripts of *Elovl3* and *Elovl6* and low-dose of L-arabinose decreased mRNA levels of *Srebf1, Fasn* and *Elovl3* (Figure 5E); protein level of cleaved SREBP1 was increased by HFD (Figure 5F). These data suggest that HFD could enhance synthesis of especially very long chain fatty acids in WAT and L-arabinose inhibit lipid synthesis in WAT by downregulating key enzymes which contribute to its alleviation of HFD-induced MS. In addition, HFD upregulated mRNA level of *Ap2* in WAT (Figure 5E), which might contribute to HFD-induced increase of both WAT weight and serum pro-inflammatory cytokines. mRNA level of leptin was also potently increased by HFD in WAT (Figure 5E), though not as dramatically as in BAT.

In heart, in regard to lipid catabolism, HFD significantly increased the levels of mRNA transcripts of *Cpt1, Cpt2, Pparα, Pparγ* and *Acadm and* L-arabinose recovered mRNA level of *Cpt1, Cpt2, Pparγ* and *Acadm;* besides, L-arabinose robustly increased mRNA level of *Ucp3* in mice on HFD (Figure 5G); while HFD increased protein level of CPT1A and UCP3 and L-arabinose treatment further increased protein level of CPT1A (Figure 5H). These data suggest that HFD promotes fatty acid transportation into the mitochondria, enhance the subsequent fatty acid beta-oxidation and increases thermogenesis by upregulating key molecules in heart, probably due to adaptation to sufficient availability of circulating lipids; and the levels of these key molecules in heart recover with L-arabinose treatment possibly because L-arabinose restores serum lipids. On the other hand, in regard to lipid anabolism, HFD did not affect the mRNA levels of the tested genes though increased protein level of cleaved-SREBP1(Figure 5G,H); while both low-dose and high dose of L-arabinose treatment reduced mRNA levels of *Elovl6* and *Hmgcr* (3-hydroxy-3-methylglutaryl-Coenzyme A reductase) (Figure 5G), which is the rate-limiting enzyme in cholesterol biosynthesis, suggesting L-arabinose suppresses elongation of long-chain fatty acid and cholesterol biosynthesis in heart of mice on HFD.

In muscle, in regard to lipid catabolism, HFD increased mRNA levels of *Cpt1* and *Ucp3 and* L-arabinose decreased mRNA level of *Pparα* (Figure 5I); while HFD decreased protein level of CPT1A, PPARα and UCP1; L-arabinose recovered CPT1A level and robustly increased UCP3 level (Figure 5J). In regard to lipid anabolism, HFD decreased mRNA levels of *Elovl6* and *Hmgcr* (Figure 5I) as well as protein level of SREBP1 (Figure 5J) and L-arabinose increased mRNA levels of *Hmgcr* and *Acox1* (acyl-Coenzyme A oxidase 1) (Figure 5I), which catalyzes the desaturation of medium chain fatty acyl-CoAs. Notably, L-arabinose significantly substantially decreased protein level of cleaved-SREBP1 (Figure 5J). These data suggest L-arabinose might increase fatty acid transportation into the mitochondria and thermogenesis and simultaneously inhibit lipid synthesis in muscle of mice on HFD, which could contribute to its amelioration of HFD-induced MS.

Taken together, in those tissues actively metabolizing lipids, both lipid catabolism and anabolism altered in response to HFD and L-arabinose treatment; L-arabinose’s alleviation of HFD-induced MS might be mediated by upregulating expression of genes involved in lipid utilization and thermogenesis markers on one hand and on the other downregulating expression of genes involved in lipid synthesis. Remarkably, the modulation of leptin expression in BAT by HFD and L-arabinose treatment should play a key role in the alterations of circulating leptin level.

### 3.6. L-arabinose Protects Liver and Heart Mitochondrial Function from HFD-Induced Damage and Improves Mitochondrial Function in Muscle in Mice on HFD 

**Figure 6:**
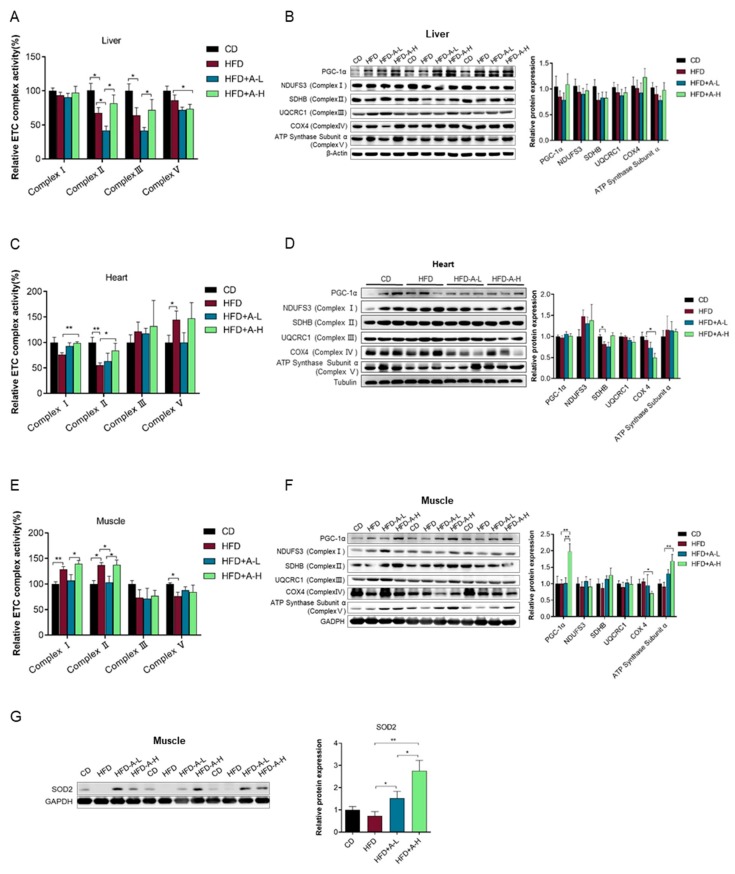
Alterations of mitochondrial ETC complexes activities and mitochondrial protein levels in liver, heart and muscle of mice among the four experimental groups. Relative activities of mitochondrial ETC (electron transport chain) complexes in liver tissue (**A**), heart tissue (**C**) and muscle tissue (**E**). Protein expression of PGC1-α and indicated subunits of mitochondrial ETC complexes were detected by SDS-PAGE and W.B. and quantified by densitometry analysis in liver tissue (**B**), heart tissue (**D**) and muscle tissue (**F**). (**G**) Protein expression of SOD2 detected by SDS-PAGE and W.B. in muscle tissue, representative blot image (left) and densitometry quantification (right) is shown. (**A**), CD, *n* ≥ 10; HFD, *n* ≥ 11; HFD+A-L, *n* = 6; HFD-A-H, *n* = 6; (**B**), CD, *n* = 6; HFD, *n* = 6; HFD+A-L, *n* = 6; HFD-A-H, *n* ≥ 5; (**C**) or (**D**), CD, *n* = 6; HFD, *n* = 6; HFD+A-L, *n* = 6; HFD-A-H, *n* = 3; (**E**), CD, *n* = 6; HFD, *n* = 6; HFD+A-L, *n* = 5; HFD-A-H, *n* = 5; (**F**), CD, *n* = 6; HFD, *n* = 6; HFD+A-L, *n* ≥ 5; HFD-A-H, *n* ≥ 5 ; (**G**), CD, *n* = 6; HFD, *n* = 6; HFD+A-L, *n* = 6; HFD-A-H, *n* = 6. Values are mean ± SEM. Statistical analyses were conducted using two-tailed unpaired *t*-test analysis. * *p* < 0.05, ** *p* < 0.01, *** *p* < 0.001.

Mitochondrial metabolism has been proposed to tune insulin action via redox-related modulation of insulin receptor or insulin receptor substrates mainly involving NAD^+^/NADH ratio and ROS (reactive oxygen species), meanwhile insulin signaling could in turn regulate mitochondrial metabolism through facilitating mitochondrial biogenesis and stimulating mitochondrial oxidative capacity and ATP production in multiple tissues [44]. The above results have shown that L-arabinose treatment improves insulin responsiveness impaired by HFD. We thereby wondered whether and how activities and expression levels of mitochondrial electron transport chain (ETC) complexes were altered among the four experimental groups. We looked into several selected tissues that are most-active in energy metabolism including liver, heart and muscle. We found that HFD significantly decreased the activities of ETC Complex Ⅱ and Complex Ⅲ in liver compared with CD and L-arabinose treatment decreased activities of Complex Ⅱ and Complex Ⅴ in liver of mice on HFD (Figure 6A) with the protein levels of the master regulator of mitochondrial biogenesis PGC1-α and of all five ETC complexes unaltered among four experimental groups (Figure 6B). In heart, the activity of ETC Complex II was also significantly impaired by HFD but importantly was successfully restored by high dose of L-arabinose treatment; additionally, high dose of L-arabinose increased the activity of Complex Ⅰ in mice on HFD, though HFD only exhibited a tendency of decreasing it; and the activity of Complex Ⅴ was interestingly increased by HFD in heart of mice (Figure 6C); while protein levels of PGC1-α and all five ETC complexes remained similar except that Complex II was lower in mice on HFD and Complex Ⅳ was decreased by high dose of L-arabinose treatment in mice on HFD (Figure 6D). Nevertheless, in skeletal muscle, HFD interestingly increased the activities of ETC Complex I and Complex II but decreased the activity of Complex Ⅴ, low-dose of L-arabinose abolished the effect of HFD on muscle Complex Ⅱ activity but high dose of L-arabinose increased the activities of both Complex I and Complex II compared with low-dose (Figure 6E). This peculiar phenomenon might involve complicated factors with different mechanisms underlying the increase of complexes activities induced by HFD and by high dose of L-arabinose, respectively. Remarkably, we observed a robust increase in PGC1-α and ETC Complex V protein level induced by high dose of L-arabinose treatment in mice on HFD (Figure 6F), suggesting L-arabinose might promote mitochondrial biogenesis and the capacity for ATP synthesis in skeletal muscle. We also checked the protein level of the most important antioxidant enzyme localized in mitochondria, SOD2 and also observed a robust increase induced by L-arabinose treatment especially high dose of L-arabinose in mice on HFD (Figure 6G), indicating that L-arabinose could enhance endogenous mitochondrial antioxidant defense to maintain redox homeostasis in skeletal muscle which is often perturbed in HFD model. These data suggest that the alleviation of metabolic syndrome in mice on HFD could be mediated via protecting mitochondrial ETC complexes function from HFD-induced disorders especially in heart and particularly promoting expression of mitochondrial ATP synthase and SOD2 in skeletal muscle.

### 3.7. Antibiotics Abolishes Hydrogen Production in Response to L-Arabinose Gavage in Both CD and HFD Fed Mice

**Figure 7:**
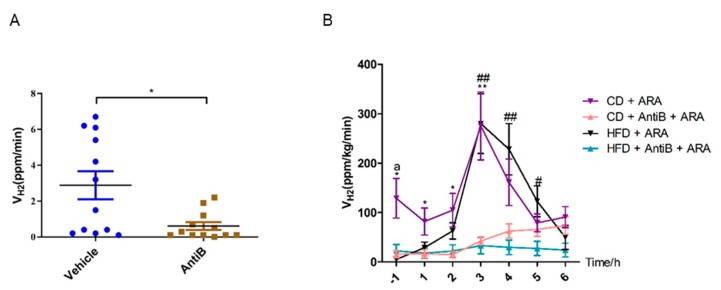
Antibiotics abolishes hydrogen production in response to L-arabinose gavage both in mice on CD and on HFD. Mice on CD were randomly assigned into two groups and were administered a combination of antibiotics, AntiB or vehicle via oral gavage daily for 3 days and then hydrogen production rate of these mice was measured using the self-built device (**A**) (*n* = 12). Then, each group of mice were sub-divided in to two groups and fed CD or HFD, respectively. All mice were given oral arabinose (5 g/kg) for 1 week. Then, hydrogen production rate were measured 1 hour before and every hour up to 6 hours after L-arabinose gavage; L-arabinose was administered via oral gavage at 0 time-point (**B**) (CD + antiB + ARA, *n* = 12; CD + ARA, *n* = 12; HFD + antiB + ARA, *n* = 10; HFD + ARA, *n* = 10). Values are mean ± SEM. Statistical analyses were conducted using two-tailed unpaired *t*-test analysis. (**A**) * *p* < 0.05. (**B**) * *p* < 0.05, ** *p* < 0.01, *** *p* < 0.001, CD + ARA vs. CD + AntiB + ARA; ^#^ *p* < 0.05, ^##^ *p* < 0.01, ^###^ *p* < 0.001, HFD + ARA vs. HFD + AntiB + ARA; ^a^ *p* < 0.05, ^aa^ *p* < 0.01, ^aaa^ *p* < 0.001, CD + ARA vs. HFD + ARA.

Hydrogen gas or molecular hydrogen, can be produced by gut flora. In order to ascertain whether gut microbe was involved in the rapid and robust hydrogen production led by L-arabinose gavage, we fed a separate cohort of mice and administered 200 µL volume of vehicle or a combination of 5 antibiotics composed of neomycin (10 mg/mL), gentamicin (5 mg/mL), ampicillin (10 mg/mL), metronidazole (10 mg/mL) and vancomycin (2.5 mg/mL) by oral gavage once a day for 3 days. The combo antibiotics cover a wide spectrum of bacteria and can suppress most gut/intestinal bacteria to extremely low levels. Then we measured the hydrogen production velocity and detected extremely low level of hydrogen release in these new batch of mice, which is consistent with the finding of Figure 1B. even though the background hydrogen production capacity is very limited, antibiotics treatment still significantly repressed the hydrogen production rate (Figure 7A). We next put these mice either on CD or HFD, changed the antibiotics delivery method from oral gavage to supplementing antibiotics to drinking water at one tenth of the concentrations in the gavage solution (neomycin (1 g/L), gentamicin (0.5 g/L), ampicillin (1 g/L), metronidazole (1 g/L) and vancomycin (0.25 g/L)) and treated mice with water or high dose of L-arabinose by oral gavage once every day for 1 week. After that, we measured the hydrogen production velocity again and strikingly we found that the robust hydrogen production following L-arabinose gavage was abolished in antibiotics treated mice independent of diet type (Figure 7B), indicating that the rapid and robust hydrogen production led by L-arabinose gavage is derived from gut microbe and gut microbe is necessary for hydrogen gas production induced by L-arabinose. Besides, although L-arabinose elicited rapid as well as robust production of hydrogen gas in mice on HFD and mice on CD, 1-week of L-arabinose treatment resulted in a substantially higher level of hydrogen production velocity before L-arabinose gavage in mice on CD compared with mice on HFD (Figure 7B) and hydrogen production velocity 4–6 hours after L-arabinose gavage is slightly higher in antibiotics treated mice on CD compared with antibiotics treated mice on HFD (Figure 7B), suggesting that HFD might have some inhibitory effects on hydrogen yield of gut microbes.

### 3.8. L-arabinose Modulates Relative Abundances of Hydrogen-Producing and Hydrogen-Consuming Gut Microbes in Mice on HFD

**Figure 8:**
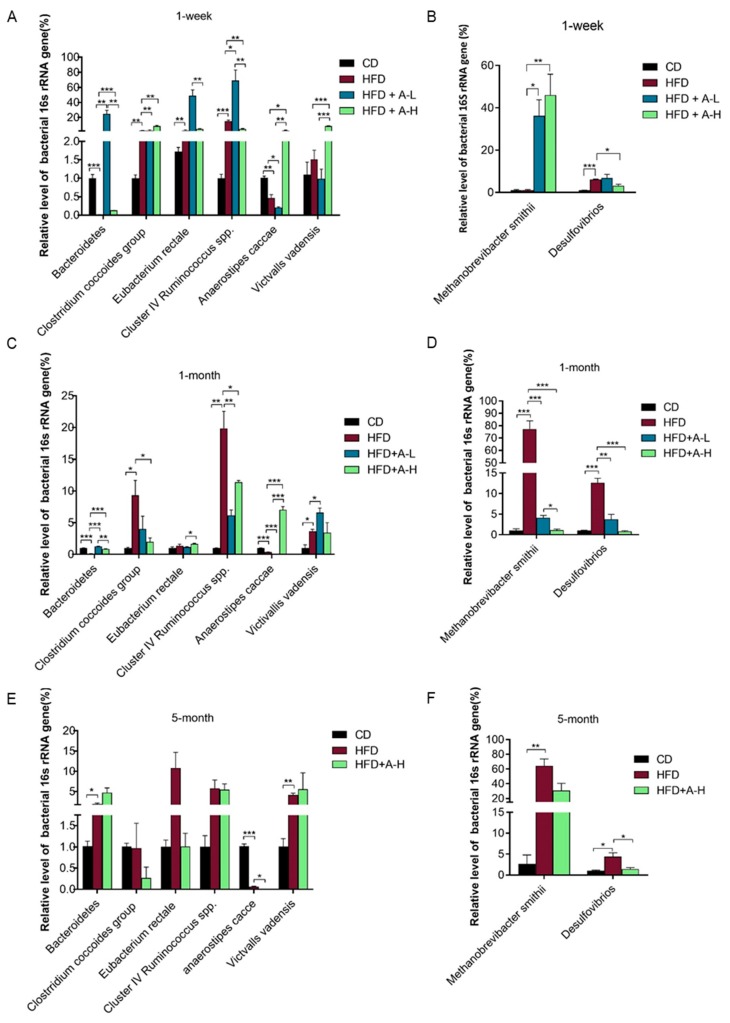
Alterations of relative abundances of hydrogen-producing and hydrogen-consuming gut microbes among the four experimental groups at 1-week, 1-month and 5-month time-points. Relative abundances of indicated gut microbes involved in hydrogen production (**A**,**C,E**) or hydrogen consumption (**B**,**D**,**F**) were determined by real-time q-PCR using primers targeting specific bacterial 16s rRNA gene at 1-week (**A**,**B**), 1-month (**C**,**D**), 5-month (**E**,**F**) time-points, respectively. Values are mean ± SEM (*n* = 3). Statistical analyses were conducted using two-tailed unpaired *t*-test analysis. * *p* < 0.05, ** *p* < 0.01, *** *p* < 0.001.

Hydrogen gas yield in the gut depends on the net result of the balance between the activities of hydrogen-producing and hydrogen-consuming microbes [23]. To corroborate and further elucidate the mechanisms of hydrogen gas production elicited by L-arabinose gavage, we detected the relative abundance of certain genera, species or groups of gut microbes with reported roles in hydrogen production or consumption by q-PCR employing primer-sets targeting specific variable regions of 16S ribosomal RNA gene sequences (see Table 3 for primer sequences). Overall, 6 pairs of primer-sets targeting different hydrogen-producing microbes and 2 pairs of primer-sets targeting different hydrogen-consuming microbes (methane-producing and sulfate-reducing, respectively) were used in the analysis. We found both diet type and L-arabinose treatment affected the relative abundances of these microbes and the effects altered through time course (Figure 8). At 1-week time-point, among all tested hydrogen-producing microbes, HFD stimulated the proliferation of *Clostridium coccoides* group, species *Eubacterium rectale* and Cluster Ⅳ *Ruminococcus* spp. while lowered relative abundances of genus *Bacteroidetes* and species *Anaerostipes caccae* and L-arabinose of either high dose or low-dose increased relative abundances of genus *Bacteroidetes, Clostridium coccoides* group, Cluster Ⅳ *Ruminococcus* spp., species *Anaerostipes caccae and* species *Victivallis vadensis* (Figure 8A); among the two tested hydrogen-consuming microbes, L-arabinose treatment, of both high dose and low-dose, increased relative abundance of hydrogen-utilizing-methane-producing archaeon species *Methanobrevibacter smithii,* which was not affected by HFD in comparison with CD and low-dose of L-arabinose treatment decreased relative abundance of hydrogen-utilizing-sulfate-reducing genus *Desulfovibrios* which was increased by HFD compared to CD (Figure 8B). At 1-month time-point, among all tested hydrogen-producing microbes, HFD increased relative abundances of *Clostridium coccoides* group, Cluster Ⅳ *Ruminococcus* spp. and species *Victivallis vadensis* while lowered relative abundances of genus *Bacteroidetes* and species *Anaerostipes caccae* and L-arabinose treatment of either high dose or low-dose effectively restored relative abundances of those microbes except for species *Victivallis vadensis* (Figure 8C); among the two tested hydrogen-consuming microbes, L-arabinose treatment of both high dose and low-dose substantially restored relative abundances of both microbes which were both dramatically lifted by HFD compared with CD (Figure 8D). At 5-month time-point, among hydrogen-producing microbes, HFD lifted relative abundances of genus *Bacteroidetes* and species *Victivallis vadensis* and lowered relative abundance of species *Anaerostipes caccae* and high dose of L-arabinose furthered decreased relative abundance of species *Anaerostipes caccae* (Figure 8E); meanwhile, HFD significantly increased relative abundances of both two tested hydrogen-consuming microbes and high dose of L-arabinose treatment significantly restored relative abundances of genus *Desulfovibrios* (Figure 8F). These data indicate that gut microbes participating in hydrogen-cycling drastically and immediately proliferate upon L-arabinose treatment and keep remodeling throughout the 5-month experimental time course. The observed increase of hydrogen release after L-arabinose gavage at three time-points suggest that there was more hydrogen produced than consumed in the gut lumen within the experimental time course. Therefore, we speculate that overall the activities of hydrogen-producing microbes should run over the activities of hydrogen-consuming microbes. L-arabinose may directly stimulate the proliferation of hydrogen-producing microbes as it could serve as a substrate of fermentation for these microbes, whereas the proliferation of hydrogen-consuming microbes might only owe to increased availability of hydrogen in gut niche therefore represent secondary effect of L-arabinose. Obviously, the balance between the two microbial groups in response to L-arabinose eventually determined a net yield of hydrogen gas at all three time-points, though the hydrogen yield attenuate over time. According to a recent study in human, hydrogen production capacity of the gut microbes seems to alter with age, in particular decreases with age in males [45], which might provide a possible explanation to our results that age, at least in part, accounted for the decline of the hydrogen yield.

HFD seemed to stimulate proliferation of gut microbes participating in hydrogen-cycling too, especially after long time feeding. However, considerable hydrogen release was not detected in mice on HFD at any of the three time-points (Figure 1B–D). Notably, the relative abundances of hydrogen-consuming microbes in HFD group were much higher compared to those in L-arabinose groups at both 1-month and 5-month time-points (Figure 8D,F), especially at 1-month time-point the relative abundance of *Methanobrevibacter smithii* in HFD group was one to two orders of magnitude higher than that in L-arabinose groups (Figure 8D). Therefore, we speculate that HFD exert equivalent stimulating force on hydrogen-producing and hydrogen-consuming microbes, which eventually yield no net hydrogen production. Nevertheless, L-arabinose treatment on top of HFD robustly inhibited the proliferation of hydrogen-consuming microbes at both 1-month and 5-month time-points (Figure 8D,E), which should directly contribute to the net yield of hydrogen gas in those mice.

### 3.9. L-arabinose Modulates Relative Abundances of Gut Bacteria Involved in Metabolic Syndrome

**Figure 9:**
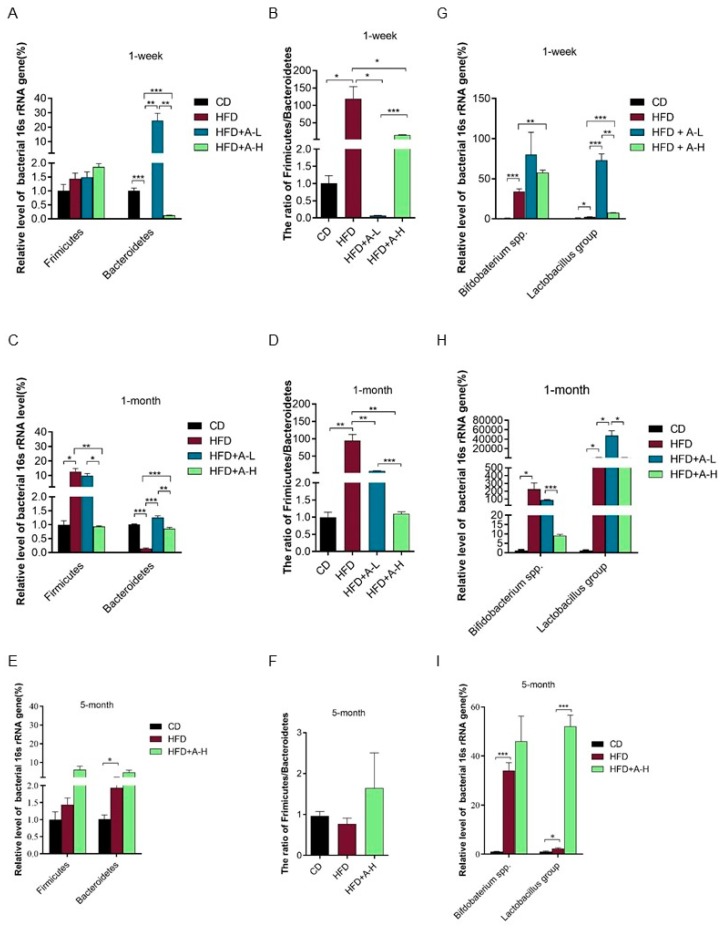
Alterations of relative abundances of gut bacteria involved in metabolic syndrome among the four experimental groups at 1-week, 1-month and 5-month time-points. Relative abundances of indicated gut microbes were determined by real-time q-PCR using primers targeting specific bacterial 16s rRNA gene at 1-week (**A,G**), 1-month (**C,H**), 5-month (**E,I**) time-points, respectively. The ratio of relative abundances of Firmicutes to Bacteroidetes at 1-week (**B**), 1-month (**D**) and 5-month (**F**) time-points were respectively calculated. Values are mean ± SEM (*n* = 3). Statistical analyses were conducted using two-tailed unpaired *t-*test analysis. * *p* < 0.05, ** *p* < 0.01, *** *p* < 0.001.

The *Bacteroidetes* and the *Firmicutes* are the two dominant groups of beneficial gut microbes and the relative proportions of *Firmicutes* and *Bacteroidetes* have been found to be positively and negatively correlated with obesity, respectively, in both human and mice [46]. Manipulating the ratio of *Firmicutes* to *Bacteroidetes* can cause weight loss or weight gain in mice [47]. We also examined the changes of the relative abundance of *Firmicutes* and *Bacteroidetes and* calculated the ratio of *Firmicutes* to *Bacteroidetes* in the feces of these mice at three time-points. At 1-week time-point, relative abundance of *Firmicutes* was not altered among four groups, while relative abundance of *Bacteroidetes* was decreased markedly by HFD compared with CD and was significantly increased by both low-dose and high dose of L-arabinose treatment, especially was dramatically boosted by low-dose of L-arbinose (Figure 9A). More strikingly, the ratio of *Firmicutes* to *Bacteroidetes* in the feces of mice on HFD was elevated approximately 100 times compared to CD and was effectively restored by both high dose and low-dose of L-arabinose (Figure 9B). At 1-month time-point, relative abundance of *Firmicutes* in the feces of mice on HFD was remarkably lifted compared with CD and was restored to the same level as CD by high dose of L-arabinose and relative abundance of *Bacteroidetes* in feces was largely restrained in mice on HFD but was restored to normal level as in mice on CD by both high dose and low-dose of L-arabinose (Figure 9C). Similarly, the ratio of *Firmicutes* to *Bacteroidetes* was drastically elevated (approximately 100 times) in the feces of mice on HFD compared with CD and was restored by both high dose and low-dose of L-arabinose; notably, high dose of L-arabinose treatment brought *Firmicutes* to *Bacteroidetes* ratio in mice on HFD to the same level as mice on CD (Figure 9D). However, at 5-month time-point, no significant alterations were brought in by HFD or L-arabinose treatment in relative abundance of *Bacteroidetes* or *Firmicutes* or the ratio of *Firmicutes* to *Bacteroidetes*, except that HFD lifted relative abundance of *Bacteroidetes* (Figure 9E,F). Notably, high dose and low-dose of L-arabinose exhibited significantly different effects on modulation of relative abundances of these two groups of beneficial gut microbes and their ratio. Overall, these findings indicate that L-arabinose can restore the relative proportion of *Firmicutes* and *Bacteroidetes* as well as *Firmicutes* to *Bacteroidetes* ratio in mice feces perturbed by HFD, which might be one of the mechanisms underlying L-arabinose’s beneficial effects in MS induced by HFD.

In addition, plenty of evidence has proved that probiotics, mainly the *Lactobacillus* strains and *Bifidobacterium* strains, in both mice on HFD and humans with metabolic disorders, can exert multiple favorable effects, including reducing body weight and fat accumulation, improving insulin resistance and glycemic control, ameliorating lipid profile, anti-inflammation and preventing non-alcoholic fatty liver disease (NAFLD) [48]. We detected the relative abundances of *Bifidobacterium* spp. and *Lactobacillus* group in the feces of these mice at the three time-points. HFD intensively increased the relative abundance of *Bifidobacterium* spp. at all three time-points with around 200-fold increase at 1-month time-point (Figure 9G,H,I). L-arabinose further increased the relative abundance of *Bifidobacterium* spp. at 1-week time-point and showed a tendency of further increasing it at 5-month time-point in mice on HFD (Figure 9G,I). Meanwhile, the relative abundance of *Lactobacillus* group was also significantly raised by HFD at all three time-points, especially was drastically raised by HFD at 1-month time-point with around 1000-fold increase in HFD group compared to CD group; and L-arabinose treatment, of either low-dose or high dose, tremendously further increased it at all three time-points (Figure 9G,H,I). These findings suggest that both *Bifidobacterium* spp. and *Lactobacillus* group immediately and continuously proliferate upon HFD feeding, perhaps in an adaptive effort to prevent MS development induced by HFD and L-arabinose’s MS-ameliorating effects could be mediated by stimulating proliferation of probiotic strains of *Bifidobacterium* spp. and *Lactobacillus* group.

## 4. Discussion

In the current study, we corroborated L-arabinose’s efficacy in HFD-induced MS, specifically in reducing body weight gain and fat weight, improving insulin sensitivity, alleviating liver steatosis and dyslipidemia and decreasing serum inflammatory cytokines in mice on HFD. Further, we assessed expression profile of key lipid metabolism genes as well as mitochondrial function in several metabolic tissues and identified some potential targets modulated by L-arabinose. Importantly, we observed a dose-and-time-dependent robust release of hydrogen gas in mice after L-arabinose gavage, which could be abolished by antibiotics treatment independent of diet type, indicating gut microbes as the source of hydrogen gas; q-PCR analysis of fecal 16s rRNA gene revealed that HFD and L-arabinose alters relative abundances of both hydrogen-producing and hydrogen-consuming gut microbes as well as some probiotics. Remodeling balance between hydrogen-producing and hydrogen-consuming gut bacteria as well as modulating relative abundances of probiotics could be the mechanism underlying L-arabinose’s eliciting hydrogen release, which might subsequently modulate lipid metabolism gene expression and mitochondrial function in metabolic tissues; and synergistically these factors add up and eventually improve multiple parameters in HFD-induced MS.

Molecular hydrogen or hydrogen gas, has been demonstrated to be efficacious in prevention and treatment against many diseases related to oxidative stress in both model animal experiments and clinical examinations [15,16,17,29], including MS [25,26,31,49]. There are several ways for hydrogen supplementation, inhaling H_2_ gas, injecting saline with dissolved H_2_ or drinking water with dissolved H_2_ (H_2_-water). Our study demonstrated hydrogen gas could be induced by nutrients like L-arabinose via selective proliferation of intestinal microbes or modulating balance between hydrogen production and consumption in gut niche, which could also be an ideal way for hydrogen supplementation. Endogenous hydrogen derived from gut microbiota can be substantial in amount when particular nutrients like L-arabinose are available in gut and can make a great impact on host health. In fact, L-arabinose is not the only one inducing hydrogen production. Acarbose, for example, an *α*-glucosidase inhibitor used as an anti-diabetic drug to treat type2- and prediabetes, markedly increase H_2_ production in the gastrointestinal tract, which is proposed to be attributable at least in part for its cardiovascular benefits [21]. Fructo-oligosaccharide and galactosyl-sucrose was also reported to remarkably increase breath hydrogen gas excretion due to hydrolysis by small intestinal enzymes and the subsequent fermentation in the large intestine in human [22]. Therefore, increasing endogenous hydrogen production through modulating gut microbial composition by administration of prebiotic nutrients or hydrogen-producing probiotics might be considered in development of therapeutic strategies involving molecular hydrogen against oxidative stress associated diseases.

Molecular hydrogen, which to a considerable extent determines the gas atmosphere in the gut niche, has also recently been demonstrated to participate in the regulation of bile acid metabolism, inhibition of bile acid oxidation in particular, in some gut bacteria [50]. Accumulating evidences have revealed numerous novel roles of bile acids in regulating gut microbial colonization and microbiome structure as well as host physiological process involving farnesoid-X-receptor (FXR) signaling, especially in metabolic syndrome setting [51,52,53]. Therefore, the beneficial effects of gut-derived hydrogen production induced by L-arabinose on HFD-induced metabolic disorders could also be mediated by regulating bile acid metabolism among gut microbes. Recently, non-gut microbiota are also acknowledged to be another source of endogenous H_2_ production [54], which also impact host physiology. Although the intestinal absorption of the antibiotics administered via gavage or water-drinking into blood stream is limited, we cannot fully rule out the possibility of hydrogen production derived from microbiota living at other locations upon L-arabinose gavage, which could also be abolished after antibiotics treatment. However, gut microbes should contribute to the majority of hydrogen release as L-arabinose is not easily absorbed through the intestinal wall too.

Aside from hydrogen gas, methane and hydrogen sulfide are also components of intestinal gas that play important roles in modulation of host health and disease. The hydrogen and methane breath test has been widely used in the diagnosis of small intestinal bacterial overgrowth. Notably, both methane and hydrogen sulfide can be produced by intestinal microbes while utilizing hydrogen. It is reported that for net hydrogen production, hydrogen consumption is far more important than absolute hydrogen production, considering most hydrogen produced by colonic bacteria is consumed by gut microbial activities which is determined by hydrogen tension [55]. This supports our observation that L-arabinose seems more potent in repressing hydrogen-consuming microbes than in stimulating hydrogen-producing microbes in mice on HFD while eliciting hydrogen release. Methanogens are one of the major group of gut anaerobic archaea that scavenge hydrogen and ammonia produced by other gut microbes as substrates for the generation of methane during fermentation; and the most prevalent methanogens found in human gut is *Methanobrevibacter smithii* [56]. Studies have indicated that *Methanobrevibacter smithii* contributes to caloric harvest, host metabolism and weight gain in the development of obesity [57]. At 1-month time-point of our experiment, HFD drastically increased and L-arabinose treatment dramatically decreased the relative abundance of *Methanobrevibacter smithii*, which could be vital for L-arabinose’s alleviation of MS in HFD models. *Desulfovibrios* utilize molecular hydrogen and reduce sulfate to sulfide. Although hydrogen sulfide could act as a signaling compound at low concentration, increased hydrogen sulfide production is deemed toxic as it is thought to be associated with higher permeability of the intestinal barrier, higher susceptibility to infections and colon cancer. Hydrogen sulfide induces metabolic abnormalities in epithelial cells; healthy young adults were observed to have low prevalence of *Desulfovibrios* in fecal samples compared with elderly people and infants [37]. In our study, HFD substantially increased and L-arabinose treatment significantly decreased the relative abundance of *Desulfovibrios* at all three checked time-points, which could be a key factor contributing to L-arabinose’s benefits in MS. It should be noted that we did not check the concentration of methane or hydrogen sulfide in mice-excreted gas, yet both of them could be changing in correlation with the concentration of hydrogen gas as well as the relative abundance of the responsible microbes and should be of importance in MS. It is worth future endeavor to study the homeostasis among these three gases and the participant gut microbes, their modulation by nutritional factors and their roles in MS associated with diet. Molecular hydrogen was reported to be a safe and effective anti-inflammatory in various models, possibly via scavenging ROS [58]. Our results also implicate that gut hydrogen gas induction could possibly limit the permeability of intestinal wall indirectly via suppressing *Desulfovibrios* proliferation and the subsequent hydrogen sulfide production, which could contribute to the anti-inflammation effect of gut molecular hydrogen by reducing the absorption of LPS (lipopolysaccharide) and other inflammatory mediators in gut. Whether hydrogen gas can directly modulate the permeability of intestinal wall by affecting intestinal tight junction warrants further study. Both age and gender are reported factors affecting the result of lactulose hydrogen breath test (LHBT); the odds of a positive LHBT decreased with age in males while it increased with age in females [45]. This is consistent with our observation that the release of hydrogen gas upon L-arabinose gavage decreases with time/age, considering our experimental animals are male mice. These findings together support the notion that the relative abundances and composition of gut microbiota involved in hydrogen-cycling and the consequent hydrogen gas pressure in gut lumen alter with age in a sex-dependent manner.

In our study, we analyzed the relative abundances of hydrogen-producing and hydrogen-consuming microbes by q-PCR employing group/genus-specific primer-sets targeting the 16S rRNA gene, yet analysis of the relevant metabolic pathways involving functional genes utilizing or producing hydrogen should provide a more precise perspective with higher resolution in decoding the hydrogen-cycling system, considering that some gut microbes contain both hydrogen-producing and hydrogen-utilizing genes, like *Firmicutes* [23]. Moreover, further studies probing the mechanism of gut-derived hydrogen gas should check the levels of specific genes, fluxes or the type and amount of the products in the Wood–Ljungdahl pathway, the largest carbon fixation pathway in anaerobic conditions that enables microbes to utilize electron donor such as hydrogen and electron acceptor such as carbon dioxide for biosynthesis, acetogenesis, methanogenesis, sulfate reduction and so forth [59]. Alterations in those aspects in response to induction of gut-derived hydrogen gas could provide a more comprehensive picture on the mechanisms mediating the benefits of gut hydrogen gas.

One limitation is that fecal DNA was used as the sample for 16s rRNA gene q-PCR analysis, consequently the relative abundances of microbes we got could not fully represent the real situation in different parts of the intestine. Besides, we did not check bile acids and short-chain fatty acids production in gut microbiome in response to L-arabinose treatment, which are important mediators linking gut microbiota to alleviation of MS. Finally, gnotobiotic mice model and fecal microbiota transplant could be employed to clarify the key microbes mediating the efficacy of L-arabinose in MS.

It is reported that HFD could gradually lead to elevation of serum leptin level and central leptin resistance that affected food intake differently in three stages of HFD-induced obesity development in c57 mice [60]. In our study, we also found HFD led to elevated serum leptin level and L-arabinose treatment effectively lowered serum leptin level in mice on HFD but the food intake or calorie intake was stable and similar among the four groups throughout the experimental course in general. Appetite is a balance between hunger and satiety and is under complex regulation involving a wide spectrum of peripheral and central factors from gut to hypothalamus, like ghrelin, neuropeptide Y, agouty-related peptide and so forth [61,62,63]; serum leptin level and central leptin sensitivity is only one of the many factors. It looks like the benefits of L-arabinose in metabolic phenotype have nothing to do with appetite modulation. In addition, the type and level of dietary fat can make a difference in obesogenic outcome and the expression of appetite regulating factors, highly saturated fat diet was reported to induce obesity without hyperphagia [64], which might be one reason leading to inconsistencies in whether or not food intake changes in HFD models.

We noticed that HFD upregulated some genes that promote lipid catabolism in most tested metabolic tissues, such as *Pparα, Pparγ, Cpt1, Ucp1, Acadm* and *Ucp3* and enhanced the activities of some mitochondrial ETC complexes in heart and muscle in our experiment, implicating increased energy expenditure especially increased fatty acid transport into the mitochondria, increased subsequent beta-oxidation, increased electron transport and heat dissipation induced by HFD. HFD was reported to decrease energy expenditure and expression of genes controlling lipid metabolism, mitochondrial function and skeletal system development in the adipose tissue [65]. Despite the controversy, it should be pointed out that the energy and nutritional composition of HFD could lead to different response in specific tissues. In our case, increased energy expenditure involving increased lipid catabolism should be an adaptation to increased availability of fat in diet. Similarly, the increased relative abundances of gut probiotics in mice on HFD could also be an adaptive proliferation in an effort to resist HFD-induced obesity and MS and could have played a eventually failed protective role on host health.

In conclusion, increasing endogenous hydrogen gas release by targeting gut microbes participating hydrogen-cycling with novel nutrients like L-arabinose represents an emerging, promising, efficacious and safe approach to mitigate MS. More studies are called to provide a thorough understanding to the working mechanisms of nutritional components that are effective in MS, which is essential for development of better strategies involving nutrients to combat MS.

## Figures and Tables

**Table 1:** Compositions and formulas of chow diet and high fat diet.

N/A	Chow Diet			High Fat Diet		
**Ingredient**	**gm**	**kcal**	**Calorie%**	**gm**	**kcal**	**Calorie%**
Casein, 30 Mesh	200	800	19.72	200	800	19.72
L-Cystine	3	12	0.30	3	12	0.30
Corn Starch	506.3	2024.8	49.91	0	0	0
Maltodextrin 10	125	500	12.32	125	500	12.32
Sucrose	68.8	275.2	6.78	68.8	275	6.78
Cellulose, BW200	50	0	0	50	0	0
Soybean Oil	25	225	5.55	25	225	5.55
Lard	20	180	4.44	245	2205	54.35
Mineral Mix S10026	10	0	0	10	0	0
Dicalcium Phosphate	13	0	0	13	0	0
Calcium Carbonate	5.5	0	0	5.5	0	0
Potassium Citrate, 1 H_2_O	16.5	0	0	16.5	0	0
Vitamin Mix V10001	10	40	0.99	10	40	0.99
Choline Bitartrate	2	0	0	2	0	0
FD & C Red Dye #40	0.04	0	0	0	0	0
FD & C Blue Dye #1	0.01	0	0	0.05	0	0
Total	1055.05	4057	100	773.85	4057	100
**Component**	**gm%**	**Calorie%**		**gm%**	**Calorie%**	
Protein	19.2	20		26	20	
Carbohydrate	67.3	70		26	20	
Fat	4.3	10		36	60	
**kcal/gm**	3.85			5.24		

**Table 2:** Sequences of oligonucleotide primers targeting indicated genes related to lipid metabolism.

Abbreviation	Full Gene Name	Forward/Reverse Primer Sequence
*Pparα*	peroxisome proliferator-activated receptor alpha	F: CAAGGCCTCAGGGTACCACTAC
		R: GCCGAATAGTTCGCCGAAA
*Pparγ*	peroxisome proliferator-activated receptor gamma	F: ATTGAGTGCCGAGTCTGTGG
		R: ACCTGATGGCATTGTGAGACA
*Srebf1*	sterol regulatory element binding transcription factor 1	F: CAGACTCACTGCTGCTGACA
		R: GATGGTCCCTCCACTCACCA
*Acadm*	acyl-Coenzyme A dehydrogenase, medium chain	F: GGAGTACCCGTTCCCTCTCA
		R: CCATACGCCAACTCTTCGGT
*Hmgcr*	3-hydroxy-3-methylglutaryl-Coenzyme A reductase	F: CTTGTGGAATGCCTTGTGATTG
		R: AGCCGAAGCAGCACATGAT
*Ffar4*	free fatty acid receptor 4	F: ACCAAGTCAATCGCACCCAC
		R: GTGAGACGACAAAGATGAGCC
*Fasn*	fatty acid synthase	F: GGCCCCTCTGTTAATTGGCT
		R: CGCTTGTTGGTGGACACTTG
*Elovl3*	ELOVL^a^ fatty acid elongase 3	F: ATGAATTTCTCACGCGGGTT
		R: TGTAGGTCTGGCCAACAACG
*Elovl6*	ELOVL fatty acid elongase 6	F: TCTGATGAACAAGCGAGCCA
		R: TGAAGACGGCAAGAGTCAGC
*Cpt1*	carnitine palmitoyl transferase 1	F: CATCCACGCCATACTGCT
		R: GACCTTGAAGTAACGGCCTC
*Cpt2*	carnitine palmitoyl transferase 2	F: CAGAGACAGCACTCAGACCC
		R: TTCTCCTTAGCAGCGGTGAC
*Ucp1*	uncoupling protein 1	F: CACGGGGACCTACAATGCTT
		R: ACAGTAAATGGCAGGGGACG
*Ucp3*	uncoupling protein 3	F: GATACGCCTGGGAACTGGAG
		R: GGAGCGTTCATGTATCGGGT
*Leptin*	Leptin	F: AGGATGACACCAAAACCCTC
		R: TCTTGGACAAACTCAGAATGGG
*Ap2/Fabp4*	adipocyte protein 2/fatty acid binding protein 4	F: TGGAAGCTTGTCTCCAGTGA
		R: AATCCCCATTTACGCTGATG
*Lpl*	lipoprotein lipase	F: TTCAACCACAGCAGCAAGAC
		R: TTCTCTCTTGTACAGGGCGG
*Acox1*	acyl-Coenzyme A oxidase 1	F: GGGAATTTGGCATCGCAGAC
		R: ATTGAGGCCAACAGGTTCCA
*Gapdh*	Glyceraldehyde 3-phosphate dehydrogenase	F: AGGTCGGTGTGAACGGATTTG
		R: TGTAGACCATGTAGTTGAGGTCA

a: elongation of very long chain fatty acids (ELOVL).

**Table 3:** Sequences of oligonucleotide primers and probe targeting indicated gut microbes.

Gut Microbes	Forward Primer Sequence	Reverse Primer Sequence	Reference
*Bacteroidetes*	GGARCATGTGGTTTAATTCGATGAT	AGCTGACGACAACCATGCAG	[32]
*Firmicutes*	GGAGYATGTGGTTTAATTCGAAGCA	AGCTGACGACAACCATGCAC	[32]
*Clostridium coccoides* group	AAATGACGGTACCTGACTAA	CTTTGAGTTTCATTCTTGCGAA	[33]
*Eubacterium rectale*	AAGGGAAGCAACGCTGTGAA	CGGTTAGGTCACTGGCTTC	[34]
Cluster Ⅳ *Ruminococcus* spp.	GGCGGCYTRCTGGGCTTT	CCAGGTGGATWACTTATTGTGTTAA	[35]
*Anaerostipes caccae*	TAGCCAGCATTTGAGGTGGG	CTCACGACTTCGCTTCCCTT	N/A
*Victivallis vadensis*	TAAGTTGACCGCCTGGGAAC	CCAGGTAAGGTTCTTCGCGT	N/A
*Methanobrevibacter smithii*	CCGGGTATCTAATCCGGTTC	CTCCCAGGGTAGAGGTGAAA	[36]
*Desulfovibrios*	CCGTAGATATCTGGAGGAACATCAG	ACATCTAGCATCCATCGTTTACAGC	[37]
*Bifidobacterium* spp.	CGCGTCYGGTGTGAAAG	CCCCACATCCAGCATCCA	[38]
*Lactobacillus* group	AGCAGTAGGGAATCTTCCA	CACCGCTACACATGGAG	[39]
Domain bacteria universal	TCCTACGGGAGGCAGCAGT	GGACTACCAGGGTATCTAATCCTGTT	[40]
